# Gold-Platinum Nanoparticles with Core-Shell Configuration as Efficient Oxidase-like Nanosensors for Glutathione Detection

**DOI:** 10.3390/nano12050755

**Published:** 2022-02-24

**Authors:** Javier Bonet-Aleta, Jose I. Garcia-Peiro, Silvia Irusta, Jose L. Hueso

**Affiliations:** 1 Institute of Nanoscience and Materials of Aragon (INMA), Campus Rio Ebro, CSIC-Universidad de Zaragoza, Edificio I+D, C/Poeta Mariano Esquillor, s/n, 50018 Zaragoza, Spain; jbaleta@unizar.es (J.B.-A.); joseignacio.garcia.peiro@gmail.com (J.I.G.-P.); sirusta@unizar.es (S.I.); 2 Networking Research Center in Biomaterials, Bioengineering and Nanomedicine (CIBER-BBN), Instituto de Salud Carlos III, 28029 Madrid, Spain; 3 Department of Chemical and Environmental Engineering, Campus Rio Ebro, University of Zaragoza, C/María de Luna, 3, 50018 Zaragoza, Spain

**Keywords:** nanozyme, oxidase-mimicking, gold-platinum, glutathione, sensor

## Abstract

Nanozymes, defined as nanomaterials that can mimic the catalytic activity of natural enzymes, have been widely used to develop analytical tools for biosensing. In this regard, the monitoring of glutathione (GSH), a key antioxidant biomolecule intervening in the regulation of the oxidative stress level of cells or related with Parkinson’s or mitochondrial diseases can be of great interest from the biomedical point of view. In this work, we have synthetized a gold-platinum Au@Pt nanoparticle with core-shell configuration exhibiting a remarkable oxidase-like mimicking activity towards the substrates 3,3′,5,5′-tetramethylbenzidine (TMB) and *o*-phenylenediamine (OPD). The presence of a thiol group (-SH) in the chemical structure of GSH can bind to the Au@Pt nanozyme surface to hamper the activation of O_2_ and reducing its oxidase-like activity as a function of the concentration of GSH. Herein, we exploit the loss of activity to develop an analytical methodology able to detect and quantify GSH up to µM levels. The system composed by Au@Pt and TMB demonstrates a good linear range between 0.1–1.0 µM to detect GSH levels with a limit of detection (LoD) of 34 nM.

## 1. Introduction

In recent years, nanozymes have abruptly emerged as promising nanomaterials mimicking the activity of natural enzymes in the field of biology, medicine or sensing due to their intrinsic advantages: lower production cost, larger stability and easier chemical modification [1]. Oxidase-like nanozymes oxidize organic molecules using O_2_ as a final electron acceptor yielding the oxidized species and H_2_O or H_2_O_2_ depending on the electrons involved in the reaction [1]. Nanozymes composed by a plethora of different elements such as CeO_2_ [2], MnO_2_ [3], g-C_3_N_4_ [4] or MoO_3_ [5] have been evaluated as oxidase-like surrogates. However, oxidase-like nanozymes based on noble metals such as Au [6,7,8] or Pt [9,10,11] have been the most exploited ones. Shen et al. [12] shed light on the oxidase-like mechanism of noble metal based nanozymes by the use of density functional theory (DFT) calculations. The metallic surface plays a key role activating the O_2_ to further oxidize the substrate [12]. Taking into consideration the conservation of spin quantum number [13], the reaction of triple ground state of O_2_ (^3^∑ O_2_) with a closed-shell electronic configuration of organic substrates require a triplet transition state which are typically highly energetic [14]. Thus, the overall process will require high activation energies. A plausible reaction mechanism comprises the adsorption of O_2_ onto the noble-metal surface and its dissociation into O adatoms. The metal donates electron density to antibonding π* orbitals of ^3^∑ O_2_ to yield adsorbed O-adatoms with zero magnetic moment, which may react with the organic substrate without forming a highly energetic transition state while accomplishing the spin conservation rule [15]. These O adatoms can withdraw H from the organic substrates to oxidize them [12]. Thus, dissolved O_2_ must be activated by the metal surface. In this work, we have synthetized an Au@Pt nanozyme with core-shell configuration with a high-performance oxidase-mimicking activity [16,17,18].

In order to catalyze the O_2_ activation, the active sites present on the surface of the metallic nanoparticles must be accessible. Thus, surface modifications of nanozymes imply a change in their enzyme-like behavior. For instance, You et al. demonstrated the inhibition of the activity of citrate-capped Pt nanoparticles after their aggregation, due to the electrostatic interaction between heparin and the protamine present onto the Pt surface. Surface modification can be also caused by the attachment of thiol groups (-SH) present in a plethora of organic molecules. These functional groups display a considerable major nucleophilicity in comparison to their oxygenated (-OH) counterparts due to the larger size of the S3p orbital [19]. Among all noble-metals, the formation of Au-S bonds onto the nanoparticle surface has been widely reported [20]. Furthermore, it has been demonstrated the enzyme-like inhibitory effect [21]. Glutathione (GSH) is considered the major antioxidant molecule in living cells whose task is remove reactive oxygen species (ROS) produced in different scenarios in the cell [22]. This antioxidant molecule is an important biomarker for Parkinson’s disease [23], mitochondrial disease [24] or oxidative stress [25], while is currently being extensively investigated in cancer [26]. Thus, its detection and quantification are of great interest in the field of biomedicine. Considering its chemical structure, GSH is a tripeptide formed by glutamate, cysteine and glycine residues, where the -SH group from the cysteine is the responsible of reacting with ROS. However, this -SH group is susceptible to poison the surface of noble-metal based nanozymes, blocking its catalytic activity. This behavior has been also used to detect other analytes, such as ascorbic acid [27,28] or dopamine [29].

Herein, we report a novel methodology to detect GSH at the micromolar level with the aid of Au@Pt core-shell nanozymes with oxidase-like response. We take advantage of an indirect colorimetric probe based on 3,3′,5,5′-tetramethylbenzidine (TMB). We also exploit the surface interaction between GSH and the noble-metal based nanozyme. The presence of trace concentrations of GSH in solution is enough to bind to Au@Pt surface and progressively inhibit its oxidase-like activity towards TMB. We have developed and established a thorough analytical protocol to detect and quantify GSH with low limits of detection (LoD).

## 2. Materials and Methods

### 2.1. Chemicals

Gold(III) chloride hydrate (50% Au basis), platinum(IV) chloride H_2_PtCl_6_ acid solution, L-ascorbic acid (99%), Pluronic F-127 (F-127), glutathione (GSH, 98%), anhydrous sodium acetate (CH_3_COONa, 99%), 3,3′,5,5′-tetramethylbenzidine (TMB, ≥95%), *o*-phenylenediamine (OPD, 98%) and dimethyl sulfoxide (DMSO) were purchased from Sigma-Aldrich (Darmstadt, Germany) and used without further purification.

### 2.2. Synthesis of Core-Shell Au@Pt Nanozymes

Au@Pt were synthetized following a protocol adapted from the one reported by Yang et al. [17] and taking into consideration different aspects from other works published elsewhere [16,18]. In a typical synthesis, 60 mg of Pluronic F-127 were completely dissolved in 2 mL of distilled water. Then, HAuCl_4_ (1 mL, 50 mM) and H_2_PtCl_6_ (1 mL, 50 mM) were added to the previous solution to a total volume of 4 mL. The mixture was ultrasonicated for 1 min. Then, L-ascorbic acid (2 mL, 0.25 M) was added to the reaction mixture which was left under sonication for 15 min. During this time, the solution color will change from yellow to a red-garnet color. This color change is attributed to the reduction of Au^3+^ species to Au^0^. Finally, the mixture settled overnight without further stirring at room temperature to favor the growth of the Pt dendrite. The solution color turned from red-garnet to intensely black, indicating the reduction of the Pt^4+^ precursor to Pt^0^. The final product was then purified by centrifugation (7500 rpm for 7 min, two cycles, room temperature) and finally resuspended in 1 mL of miliQ H_2_O. The synthesis of these materials has been performed at the Platform of Production of Biomaterials and Nanoparticles of the NANBIOSIS ICTS, more specifically by the Nanoparticle Synthesis Unit of the CIBER in BioEngineering, Biomaterials & Nanomedicine (CIBER-BBN, Madrid, Spain).

### 2.3. Characterization Techniques

Scanning electron microscopy (SEM) images were recorded using an INSPECT 50 (5 kV) instrument (FEI, Thermo Fisher, Brno, Czech Republic). Nitrogen adsorption isotherms were measured at 77 K on a TRISTAR 3000 system (Micromeritics, Norcross, GA, USA). The samples were degassed under vacuum at 363 K for 1 h followed by 473 K for 10 h. Transmission electron microscopy (TEM) was performed using a FEI TECNAI T20 microscope (Tecnai, Eindhoven, The Netherlands) operated at 200 keV. Aberration-corrected scanning transmission electron microscopy (Cs-corrected STEM) images were acquired using a high angle annular dark field detector (HAADF) in a FEI XFEG TITAN electron microscope (FEI, Eindhoven, The Netherlands) operated at 200–300 kV. Elemental analysis was carried out with an EDAX detector in scanning mode. Samples were prepared by drop casting 3–5 µL of the NPs suspension onto a holey carbon TEM grid.

UV-vis spectra were obtained on a V67 UV–vis double beam spectrophotometer (JASCO, Madrid, Spain). X-ray photoemission spectroscopy (XPS) to analyze the surface of the nanoparticles was carried out with the aid of an AXIS Supra (Kratos Tech., Manchester, UK) using a monochromatic Al-Kα source (1486.6 eV) run at 15 kV and 15 mA. For the individual peak regions, a pass energy of 20 eV was used. Peaks analysis was performed with the Casa XPS software, using a weighted sum of Lorentzian and Gaussian components curves after Shirley background subtraction. The binding energies were referenced to the internal C 1 s (284.5 eV) standard. The composition of the material at different depths was obtained by measuring after sputtering the surface of the sample for 60 s using a gas cluster ion source (GCIS) positioned 45 degrees relative to the surface of the sample. The GCIS delivered clusters of 2000 atoms with 10 keV energy, the raster size was 2 mm. X-ray diffraction patterns were obtained on an Empyrean instrument (Malvern-PANalytical, Malvern, UK) in Bragg-Brentano configuration using CuKα radiation and equipped with a PIXcel1D detector. Nanoparticle Tracking Analysis was measured on a Nanosight 300 system (Malvern-PANalytical, Malvern, UK). Au and Pt contents were measured on a 4100 MP-AES instrument (Agilent, Madrid, Spain) after dissolving the sample in a mixture 5:1 of H_2_O:aqua regia.

### 2.4. Evaluation of the Oxidase-like Activity of Au@Pt with Different Organic Substrates

Au@Pt nanozyme was dissolved in a UV-vis quartz cuvette containing 2 mL of 0.05 M CH_3_COOH/CH_3_COONa buffer (pH = 6.00) to reach a final concentration of 0.01 mg·mL^−1^. Then, a freshly prepared TMB solution (20.8 mM in DMSO) or OPD solution (20.8 mM in H_2_O) were added to the cuvette. The absorbance at 652 nm or 450 nm was monitored in a UV-vis spectrometer for the TMB or OPD substrates, respectively. The Michaelis-Menten constant and maximum velocity were determined by Lineweaver-Burk plot:

$$\frac{1}{V_{0}}=\frac{K_{M}}{V_{\max}}\cdot \frac{1}{\left[\mathrm{Substrate}\right]}+\frac{1}{V_{\max}}$$

### 2.5. Analytical Protocol for GSH Quantification

A fresh solution of GSH 5 mM in CH_3_COOH/CH_3_COONa (0.05 M, pH = 4.00, denoted as “Buffer at pH = 4”) was prepared and labelled as “Solution A”. From Solution A, two consecutive solutions labelled as “B” and “C”, respectively, of 0.10 mM and 0.05 mM were prepared while keeping pH = 4. Different GSH concentrations (taken from Solution C) were incubated with 100 µL of Au@Pt (4.0 mg·mL^−1^) nanozyme for 10 min to ensure a complete reaction with the nanozyme surface (total volume adjusted to 1 mL). Then, 8 µL of a freshly prepared TMB solution (20.8 mM in DMSO) were added to the reaction mixture and left to react for 30 min. After that, the absorbance at 652 nm was registered in a UV-vis spectrometer. Both reactions were incubated in a Thermoshaker (TS100C, Biosan, Riga, Latvia) at 30 °C with a stirring rate of 500 rpm.

Limit of detection (LoD) of the protocol was calculated following the following equation:

$$\mathrm{LoD}=\frac{3.3\cdot s_{l.c}}{m}$$

where s_l.c_ and m the standard deviation of the residuals and the slope from the calibration curve, respectively. Standard deviation of the residuals was obtained from the regression analysis performed using Excel (Microsoft, Madrid, Spain).

## 3. Results and Discussion

### 3.1. Synthesis and Characterization of the Au@Pt Nanozymes

**Figure 1:**
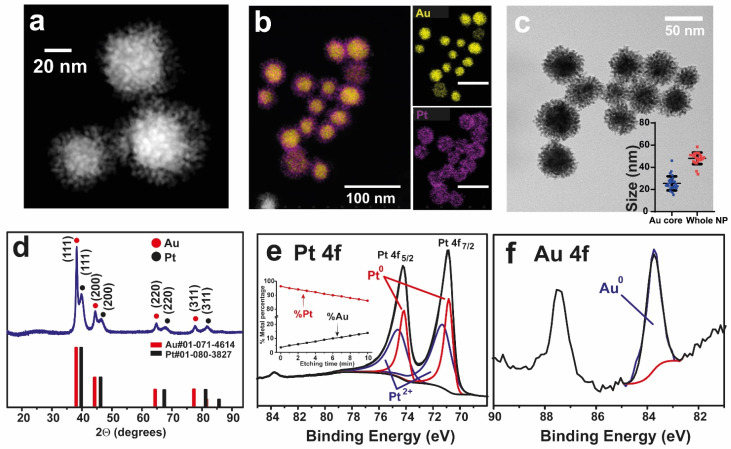
Characterization of the Au@Pt nanozymes: (**a**) HAADF-STEM image of Au@Pt nanozyme revealing the solid core and dendritic shell of the nanoparticle; (**b**) EDS mapping analysis of the Au@Pt nanoparticles based on the Au-K and Pt-L signals; Single element and overlapped mapping images suggest the presence of an Au core (depicted in yellow color) surrounded by a Pt dendritic shell (purple color) (scale bar of mapping images: 100 nm); (**c**) TEM image of Au@Pt nanozymes presenting a core/core-shell size distribution of 25.5 ± 6.3 and 48.1 ± 5.1 nm, respectively (n = 50 NPs); (**d**) X-ray Diffractogram corresponding to the Au@Pt nanozyme and its comparison with Au#01-071-4614 and Pt#01-080-3827 diffraction patterns; (**e**) X-ray photoemission spectra corresponding to the Pt4f region revealing Pt4f_5/2_ and Pt4f_7/2_ contributions associated to Pt^0^ and Pt^2+^ species. Inset: XPS depth profiling of Au@Pt nanozyme, due to core-shell nature of the nanoparticle, an increase of the etching time is correlated with the enrichment of %Au; (**f**) XPS of the Au4f region mainly consisting in a peak at BE of 83.7 eV corresponding to metallic Au.

Au@Pt nanozyme was synthetized according to methodology reported by Yang and co-workers [17]. The interplay between Au and the Pt-enriched shell is reported to play a pivotal role in the O_2_ activation and thus in the enzyme-like activity [18]. High-angle annular dark field-scanning transmission electron microscopy (HAADF-STEM) images of the Au@Pt nanoparticles revealed an interesting nanostructure consisting of a compact core surrounded by a dendritic shell revealed (Figure 1a). Scanning electron microscopy (SEM) analysis of the Au@Pt nanostructure validated its correct uniformity and dispersion (Figure S1). In terms of spatial disposition and composition, HAADF-STEM and energy dispersive X-Ray spectroscopy (EDS) mapping images confirmed a core-shell configuration where the entire core was composed by Au while the surrounding dendrite shell was made of Pt (Figure 1b). The dendritic nature of the Pt shell endorsed the nanozyme with a large specific surface area ideal to provide a greater number of active sites to fix and activate dissolved O_2_ [16,30,31,32]. The Au@Pt nanozyme fitted within a type V isotherm plot (Figure S2a) characteristic of mesoporous materials [33]. By Brunauer-Emmett-Teller (BET) analysis of AuPt (Figure S2b) a surface area of 20.6 m^2^/g was determined, which is larger than reported values for spherical nanoparticles of analogous size [34]. The internal core had an average diameter of 25.5 ± 6.3 nm while the diameter of the entire nanostructure was 48.1 ± 5.2 nm (Figure 1c). X-ray diffraction (XRD) revealed two clear independent patterns that perfectly matched with the cubic structure of Au (#01-071-4614) and Pt (#01-080-3827). This confirmed the formation of core-shell structure rather than an alloy [35,36] (Figure 1d). X-ray photoelectron spectroscopy (XPS) analysis of Pt4f (Figure 1e) indicated the coexistence of Pt^0^ and Pt^2+^. Two clear peaks located at binding energies (BEs) of 70.87 and 74.15 eV are attributed to Pt4f_7/2_ and Pt4f_5/2_ of Pt^0^, respectively [37]. This represents an energy shift of +0.44 eV in comparison to bulk Pt [38,39] due to the interaction with Au, which tends to withdraw electronic density from Pt due to its larger electronegativity (χ_Au_ = 2.53 versus χ_Pt_ = 2.28 [40]). Another two photoemission peaks at 71.33 and 74.65 eV correspond to Pt4f_7/2_ and Pt4f_5/2_ of oxidized Pt species such as PtO or Pt(OH)_2_ [37]. The BEs at 83.7 eV corresponding to the Au4f_7/2_ region, corroborated its metallic nature [41] (Figure 1f). XPS etching profiling also confirmed the core-shell configuration and revealed the increasing concentration of Au (inset in Figure 1e, see also experimental details).

The quantification of both peaks revealed a surface atomic percentage of 40% and 60% for Pt^0^ and Pt^2+^, respectively. The large percentage of oxidized Pt at the surface endorse the nanozyme with a high degree of oxophilicity [42] (i.e., tendency to bind to oxygen [43]) and thiophilicity (tendency to form S-bonds [44]). XPS depth profiling (Figure 1e, inset) further confirmed the core-shell nature of the nanozyme, since the Au at.% increased towards the inner part during the etching treatment. Hence, microwave plasma atomic emission spectroscopy (MP-AES) revealed a composition of 43% and 57% of Au and Pt in overall, respectively. Additionally, the formation of a core-shell structure was also confirmed by ultraviolet-visible spectroscopy (UV-vis) due to the appearance of a peak contribution at 620 nm corresponding to localized surface plasmon resonance (LSPR) of Au, which is shifted from conventional values of 530 nm due to the presence of Pt shell (Figure S3).

### 3.2. Oxidase-like Activity of the Au@Pt Nanozymes

**Figure 2:**
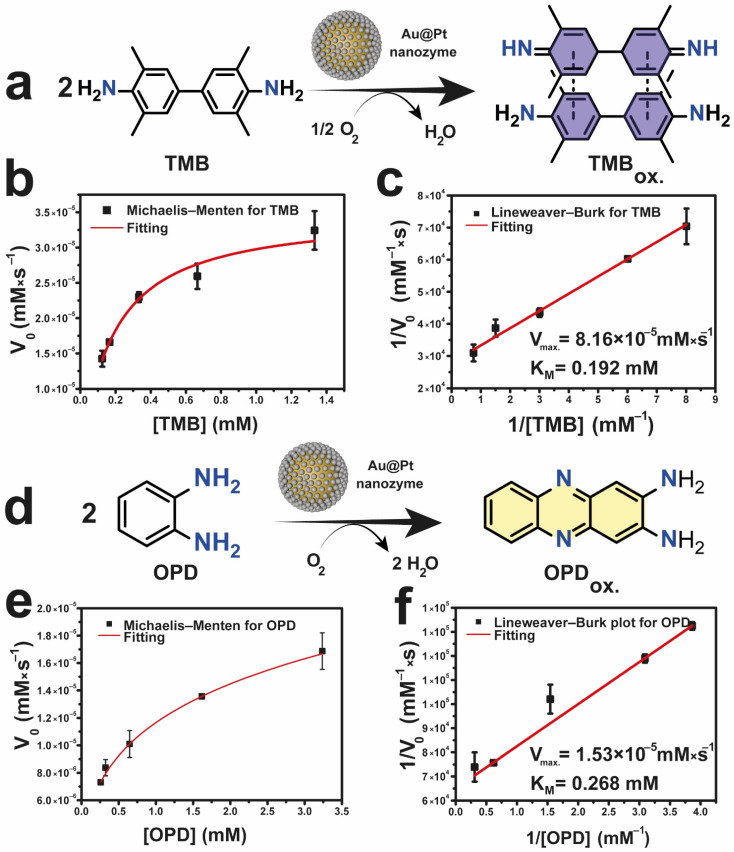
Au@Pt enzyme-like activity. (**a**) Reaction scheme of oxidation of TMB into a charge transfer complex (TMB_ox_) with a maximum absorbance at 652 nm; (**b**) Michaelis−Menten curve for Au@Pt nanozyme using TMB as substrate; (**c**) derived Lineweaver−Burk curve employed to obtain the enzymatic constants (V_max_, K_M_). The kinetic data was obtained considering the change in the absorbance at 652 nm with time; (**d**) Reaction occurred using OPD as substrate to yield yellow-colored OPD_ox_ with a maximum absorbance at 450 nm; (**e**) Michaelis−Menten curve for the Au@Pt nanozyme using OPD as substrate; (**f**) Lineweaver−Burk plot obtained from OPD oxidation used to determine enzymatic constants (V_max_, K_M_). Reaction conditions: [Au@Pt] = 0.01 mg×mL^−1^, T = 25 °C, pH = 6.00 (adjusted by using CH_3_COOH/CH_3_COONa buffer 0.05 M).

To further characterize the enzyme mimicking response of Au@Pt, we explored the catalytic oxidation of two model organic substrates: 3,3,5,5-tetramethylbenzidine (TMB) and *o*-phenylenediamine (OPD). The oxidation product of both substrates consists of a charge transfer complex (hereafter TMB_ox_) with a maximum absorption peak at 652 nm for TMB and a yellow product (hereafter OPD_ox_) with the maximum absorption peak at 450 nm (Figure 2a,d), respectively. After the addition of the nanozyme, the absorbance at 652/450 nm started to increase while in the absence of it, no changes were detected (Figure S4a,b). Moreover, the oxidase-like behavior was demonstrated by conducting the reaction after purging the solution with a N_2_ stream (Figure S5). The nanozyme activity decreased up to a 70% in the absence of O_2_. In view of these results, we assume that the Au@Pt NPs are able to activate O_2_. The most plausible activation mechanism implies the dissociation into O-adatoms. These O-atoms can subsequently withdraw an H from TMB to further oxidize it [12].

The enzyme-like behavior of Au@Pt could be fitted to a Michaelis−Menten model and linearized using the Lineweaver−Burk method (Figure 2b,c,e,f) [45]. The initial rates (V_0_, mM·s^−1^) of TMB oxidation at different TMB concentrations (mM) fitted correctly to the Michaelis-Menten equation (R^2^ = 0.98) demonstrating the enzyme-like behavior of Au@Pt (Figure 2b). The Michaelis-Menten constant (K_M_) and maximum velocity (V_max_) were determined from the Lineweaver-Burk plot (Figure 2c), adjusting the reciprocal initial velocity (V_0_^−1^, mM^−1^·s) and reciprocal concentration ([TMB]^−1^, mM^−1^) to a linear function (R^2^ = 0.99). The obtained K_M_ and V_MAX_ for TMB were determined to be 0.192 mM and 8.16×10^−5^ mM·s^−1^ respectively.

These data were calculated assuming a molar extinction coefficient (ε) for TMB_ox_ of 3.9·10^4^ L·mol^−1^·cm^−1^ [46]. Analogous results were obtained fitting the experimental data retrieved from OPD oxidation (Figure 2e) (R^2^ = 0.99) and its Lineweaver-Burk plot (Figure 2f) (R^2^ = 0.99). In this latter experiment, K_M_ and V_max_ values obtained for OPD were 0.268 mM and 1.53 × 10^−5^ mM × s^−1^, respectively. Kinetic constants were calculated assuming a ε for OPD_ox_ of 1.2 × 10^5^ L·mol^−1^ × cm^−1^ [47]. The obtained values suggested a better interaction of Au@Pt with TMB. Thus, we further selected TMB as reaction substrate to detect GSH. The lower K_M_ determined for TMB can be correlated with a better affinity with this substrate by Au@Pt [7,8,48], even more than the natural Horseradish Peroxidase (HRP) (K_M_ = 0.434 mM [49]). Moreover, the K_M_ value obtained with the Au@Pt nanodendrites is in the range of the reported values in literature for other oxidase-like nanozymes (see Table 1). In terms of V_max_, most reported values are obtained in favorable conditions (i.e., pH = 4.00). Au@Pt nanozyme offers an outstanding catalytic activity (8.16 × 10^−5^ mM × s^−1^) which compete with other oxidase-like materials also presented in Table 1. A higher V_max_ was also obtained for TMB indicating a larger reaction rate. In this regard, we expect that GSH will hinder more effectively TMB oxidation and a lower amount of GSH will be necessary to inhibit the nanozyme, which directly correlates with a major sensibility of the analysis. Au@Pt enzyme-like activity variation with concentration, temperature and pH were also studied (Figure S6). Obtained results shown a dependency of the reaction rate with the nanozyme concentration (Figure S6a,d). Au@Pt nanozyme was highly active in a wide temperature range (i.e., 25–55 °C) (Figure S6b,e), a common feature among nanozymes [50]. In terms of pH, the optimal performance was found at pH = 4.0–4.5 (Figure S6c,f) which is also the typical values reported for oxidase-like nanozymes, such as CeO_2_ or Pd [50].

### 3.3. Detection of GSH Using Au@Pt Nanozymes

**Figure 3:**
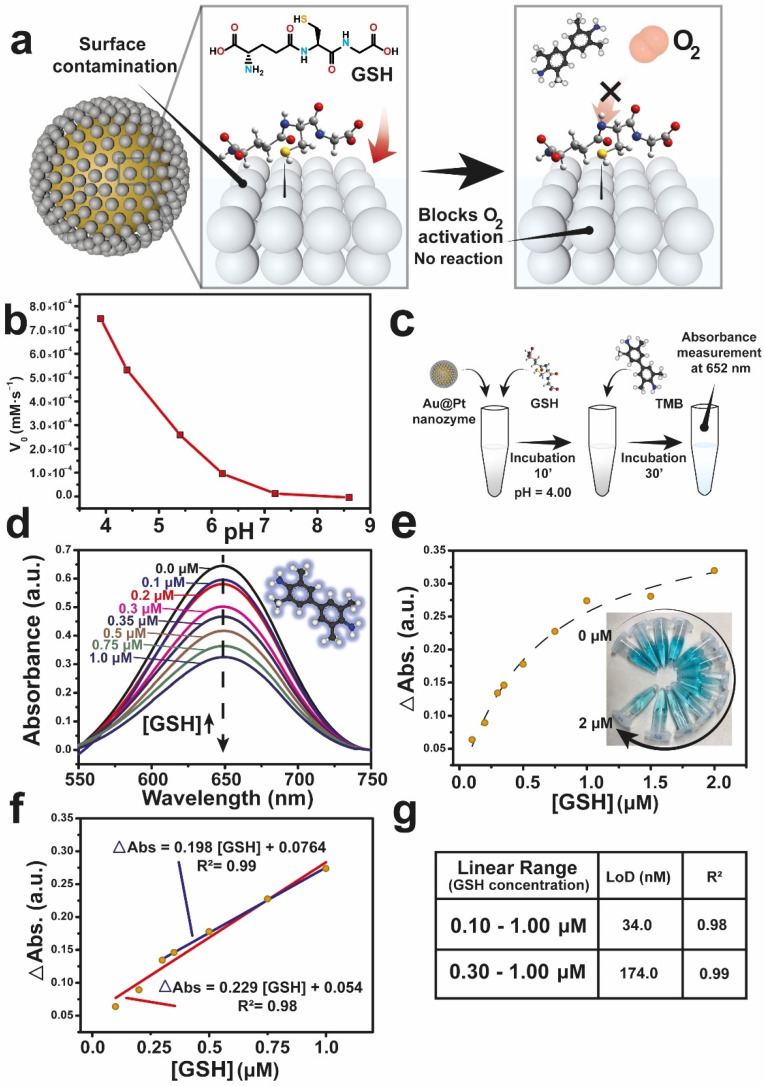
Exploiting the surface deactivation leveraged by thiol bonding to quantify GSH: (**a**) Proposed mechanism of surface deactivation of Au@Pt nanozyme by interaction of -SH group, hampering dissolved O_2_ to be activated by the metal surface that indirectly inhibits the evolution of the TMB colorimetric probe; (**b**) Oxidase-like activity of the Au@Pt nanozyme at different pH values; (**c**) Schematic illustration of the analytical protocol optimized to determine GSH, consisting in the incubation of Au@Pt+GSH for 10 min, and the subsequent incubation for 30 min with TMB to ensure a complete reaction; (**d**) Absorbance of TMB_ox_ after 30 min of reaction with increasing GSH concentrations, the insert structure accounts for the TMB structure; (**e**) ΔAbs (λ_652_) vs. GSH concentration (µM). A linear range is detected at very low GSH traces; however, as GSH concentration increases the absorbance differences between different points are smaller. Inset: digital photographs of reaction solutions after 30 min of incubation; (**f**) Calibration curves obtained from (**e**) and their respective linear fitting; (**g**) Analytical parameters (LoD and R^2^) retrieved from the analyzed linear ranges.

The enzyme-like activity of several nanomaterials is extremely sensitive to their surface properties [45]. For example, depending on their surface groups Au nanomaterials may exhibit glucose-oxidase [6,7] or peroxidase-like [21] activity. Considering the work of Shen et al. [12], the catalytic oxidation of TMB needs a O_2_ dissociation in two ·O adatoms on the metal surface. However, when GSH is present in the solution, its -SH group is able to bind to the Pt surface and prevent the O_2_ adsorption and further activation (Figure 3a). XPS analysis of Au@Pt nanozyme before and after its interaction with GSH reveal the appearance of a sulfur contribution from GSH (Figure S7a,b) at BEs of 162.5 eV. S2p peaks at higher BEs (168.5 and 166.38 eV) can be attributed to oxidized S species (S^IV^ and S^VI^) This negative shift of BE in comparison to pure GSH [54] is attributed to the bonding to a metal [55,56,57]. However, Au4f peaks do not shift after the incubation with GSH (Figure S8a), indicating a major interaction with Pt. In the poisoning process, coordinating groups of GSH, specially -SH group, are able to interact with Pt dendrite. A significant augment of the peak at higher binding energies is attributed to the Pt atoms linked to S atoms of thiolated molecule [58,59] (Figure S8b), demonstrating that the poisoned surface corresponds to Pt. By the use of colorimetric detection of TMB_ox._, we have developed an analytical protocol to quantify GSH based on its inhibitory effect. Firstly, we optimized the pH buffer to reach the maximum signal-to-noise ratio (Figure 3b). We obtained the maximum oxidase-like activity at a pH of 4, a typical trend observed for noble-metal based (Au [7] or Pt [27,28,29]) or transition-metal (Mn [60,61], Ce [2], Ni-Co [62] or Mn-Fe [51] mixed oxides) oxidase-like nanozymes. Then, the following experiments were carried out using a buffer at pH = 4.00.

Secondly, we incubated the Au@Pt nanozymes with GSH for 10 min to ensure a complete surface coverage. Then, TMB was added to the reaction and was left to incubate for 30 min. This time was thoroughly optimized to warrant the reaction completion and exclude any time dependence during the UV-vis absorbance measurements (Figure 3c). As expected, the oxidase-like activity of Au@Pt decreased with the progressive increase of the GSH concentration (Figure 3d) due to the lack of active sites to activate dissolved O_2_ and the inability to further oxidize TMB. Thus, GSH concentration can be directly related with the absorbance differences between the value obtained with/without GSH (ΔAbs). Different GSH concentrations were explored to establish a linear range (Figure 3e). From 1 µM of GSH onwards the surface of the Au@Pt nanozyme starts to be saturated not finding significant differences between different GSH concentration values. Examining the results obtained using a GSH concentration in the range of 0.1–1.0 µM, two possible calibration curves could be obtained (Figure 3f,g). According to the above results, two linear ranges were determined: 0.1–1.0 µM and 0.3–1.0 µM presented by the following equations: ΔAbs = 0.229[GSH] + 0.054 (R^2^ = 0.98) and ΔAbs = 0.198[GSH] + 0.0764 (R^2^ = 0.99), respectively. As a result, a linear correlation between the ΔAbs and the GSH concentration present in the solution could be established. The calculated limits of detection (LoDs) were 34 and 174 nM, respectively. In other terms, our analytical protocol offers a comparable and enhanced performance regarding to the LoD (34 nM) in a relatively wide linear range (0.1–1.0 µM). Comparatively, the analytical parameters (i.e., linear range and LoD) obtained from other oxidase-like nanomaterials employed to detect GSH through TMB oxidation are presented in Table 2. Given the high oxidase-like activity of Au@Pt nanozyme the linear range obtained is limited in comparison with Mn-based materials, because of the strong inhibition of the Au@Pt activity by GSH. However, this strong inhibition also favors a very low LoD, which is on the order of the lowest values reported in literature (Table 2).

## 4. Conclusions

Noble-metal based nanozymes with core-shell configuration (Au@Pt) exhibit highly active oxidase-mimicking activity, even at pHs far from the typical optimum conditions for these kinds of nanozymes. However, considering the catalytic mechanism underlying these oxidase-like materials, molecules with functional groups which can potentially bind to the nanoparticle surface hamper the O_2_ activation and thus reduce the oxidase-like activity. In this scenario, a linear correlation between the concentration of inhibitor and the observed activity can be obtained. The linear range and limit of detection obtained support the suitability of these types of metallic nanoparticles and the use of colorimetric probes as rapid, affordable and robust analytical protocols to detect biomolecules of interest in biomedical applications.

## Figures and Tables

**Table 1:** Comparison of Michaelis constant (K_M_) and maximum velocity (V_max_) for different oxidase-like nanomaterials using TMB as substrate.

Nanozyme	K_M_ (mM)	V_max_ (mM × s^−1^)	References
CeO_2_ NPs	0.80	3 × 10^−4^	[2]
Au-MSNPs	0.22	11.8 × 10^−5^	[7]
Au@Pt NCs	0.013	2.5 × 10^−4^	[9]
Citrate capped Pt NPs	0.09	7 × 10^−3^	[10]
Chitosan-Pt NPs	0.018	-	[11]
Pt@MnO_2_	0.015	1.56 × 10^−4^	[29]
MnFe_2_O_4_	0.038	3.2 × 10^−4^	[51]
Ni-Pd	0.11	2.6 × 10^−4^	[52]
Lysozyme-Pt NPs	0.630	2.7 × 10^−3^	[53]
Au@Pt nanodendrites	0.192	8.16 × 10^−5^	This work

**Table 2:** Analytical parameters obtained with different oxidase-like nanomaterials regarding colorimetric detection of GSH by using TMB as molecule probe.

Nanozyme	Linear Range (µM)	LoD (nM)	pH	References
Pt-MnO_2_	0.2–11	25	4.0	[29]
BSA-MnO_2_	0.26–26	800	3.0	[63]
MnO_2_ sheets	1.0–25	300	5.0	[64]
Mn_3_O_4_ microspheres	5.0–60	889	4.5	[65]
V_2_O_5_	0.01–0.5	2.4	5.0	[66]
Co@N-HPC	0.05–30	36	3.5	[67]
Au@Pt nanodendrites	0.1–1.0	34	4.0	This work

## Data Availability

Data available upon reasonable request to the authors.

## Supplementary Materials

The following supporting information can be downloaded at: https://www.mdpi.com/article/10.3390/nano12050755/s1: Figure S1: SEM images of the Au@Pt nanozyme; Figure S2: N_2_ adsorption isotherm and BET analysis of the Au@Pt nanozyme; Figure S3: UV-vis spectrum of the Au@Pt nanozyme; Figure S4: Influence of the nanozyme on the oxidation of TMB and OPD; Figure S5: Influence of O_2_ on the oxidase-like response of the Au@Pt nanozyme versus TMB; Figure S6: Influence of different parameters in the oxidase-like response of the Au@Pt nanozyme; Figure S7: XPS of the S2p region before and after reaction of the Au@Pt nanozyme with GSH; Figure S8: XPS of the Au4f and Pt7f regions before and after incubation with GSH.
