# Supplementary material for: Gold-Platinum Nanoparticles with Core-Shell Configuration as Efficient Oxidase-like Nanosensors for Glutathione Detection
Source: Nanomaterials (Basel). 2022 Feb 24;12(5):755. doi: 10.3390/nano12050755 (PMC8911670; doi:10.3390/nano12050755)
Supplement: Supplementary file 1 [file nanomaterials-12-00755-s001.zip › nanomaterials-1558030-supplementary.pdf]

SUPPLEMENTARY INFORMATION

Gold-Platinum Nanoparticles with Core-Shell Configuration as Efficient Oxidase-like Nanosensors for Glutathione Detection

Javier Bonet-Aleta [1,2,3], Jose I. Garcia-Peiro [1,2,3], Silvia Irusta [1,2,3] and Jose L. Hueso [1,2,3,*]

[1] Institute of Nanoscience and Materials of Aragon (INMA), Campus Rio Ebro, CSIC-Universidad de Zaragoza, Edificio I+D, C/Poeta Mariano Esquillor, s/n, 50018 Zaragoza, Spain; jbaleta@unizar.es (J.B.-A.); joseignacio.garcia.peiro@gmail.com (J.I.G.-P.); sirusta@unizar.es (S.I.)
[2] Networking Research Center in Biomaterials, Bioengineering and Nanomedicine (CIBER-BBN), Instituto de Salud Carlos III, 28029 Madrid, Spain
[3] Department of Chemical and Environmental Engineering, Campus Rio Ebro, University of Zaragoza, C/María de Luna, 3, 50018, Zaragoza, Spain
* Correspondence: jlhueso@unizar.es

SUPPLEMENTARY FIGURES

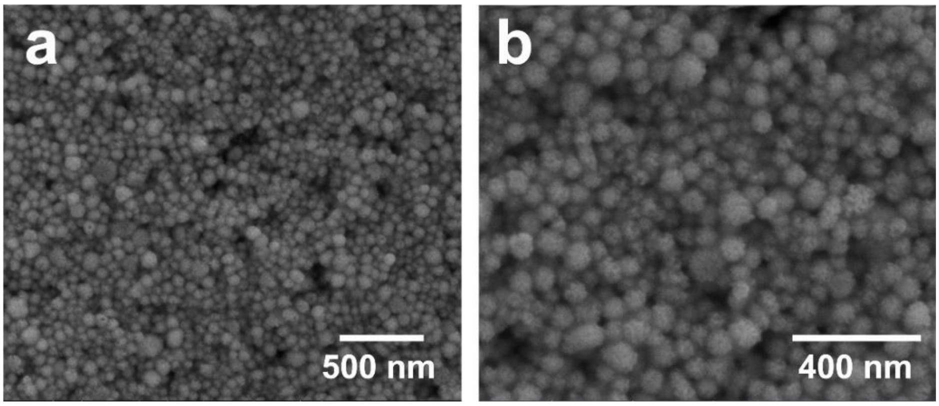

Figure S1. Scanning Electron Microscopy (SEM) images of the Au@Pt nanozyme at different magnifications.

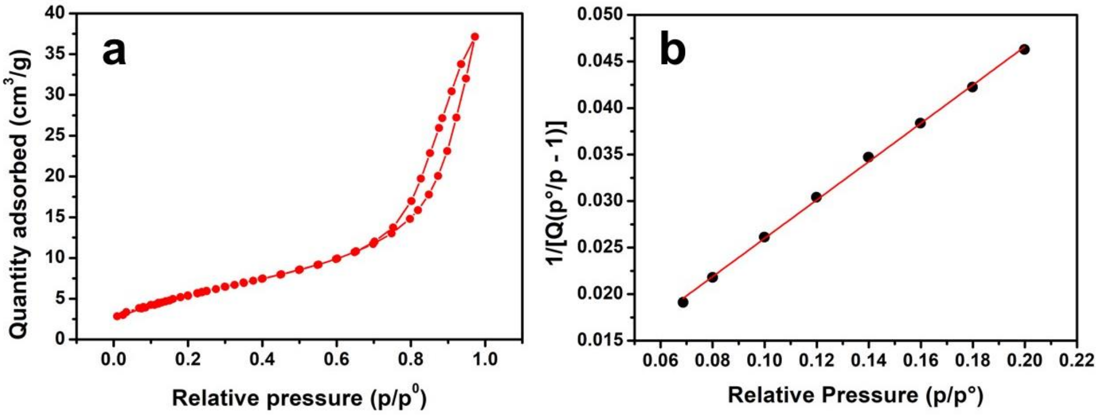

Figure S2. (**a**) N_2 adsorption isotherm at 77 K and (**b**) Brunauer–Emmett-Teller (BET) analysis of the Au@Pt nanozyme.

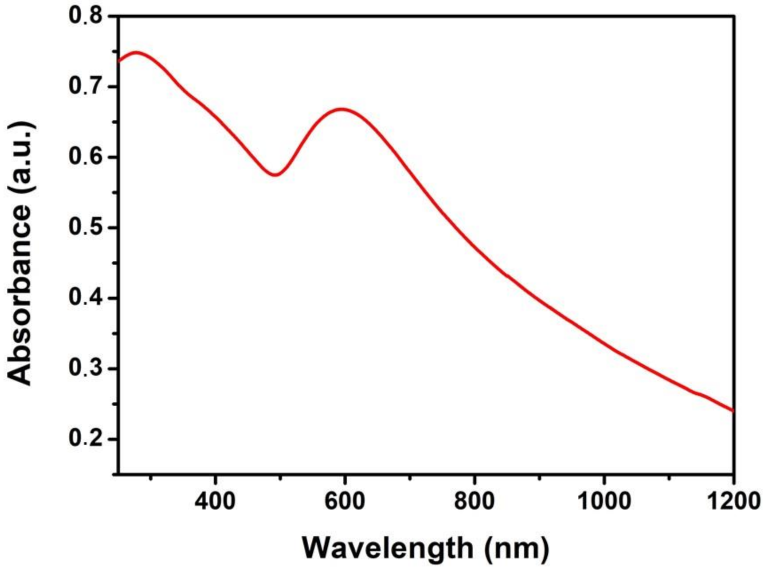

Figure S3. UV-vis spectrum of the Au@Pt nanozyme.

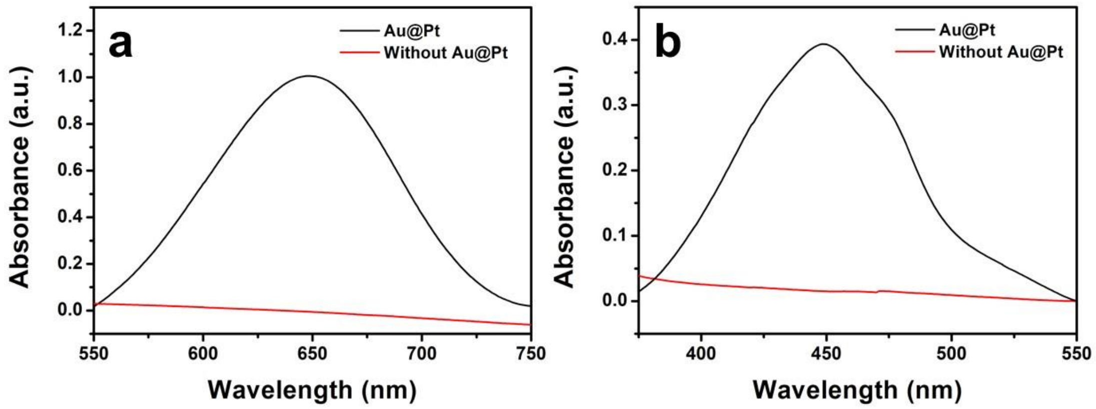

Figure S4. UV-vis spectra to evaluate the oxidation of (**a**) TMB and (**b**) OPD substrates in the absence (control experiment) and in the presence of the Au@Pt nanozymes; Reaction time in the control experiment was fixed at 1 hour.

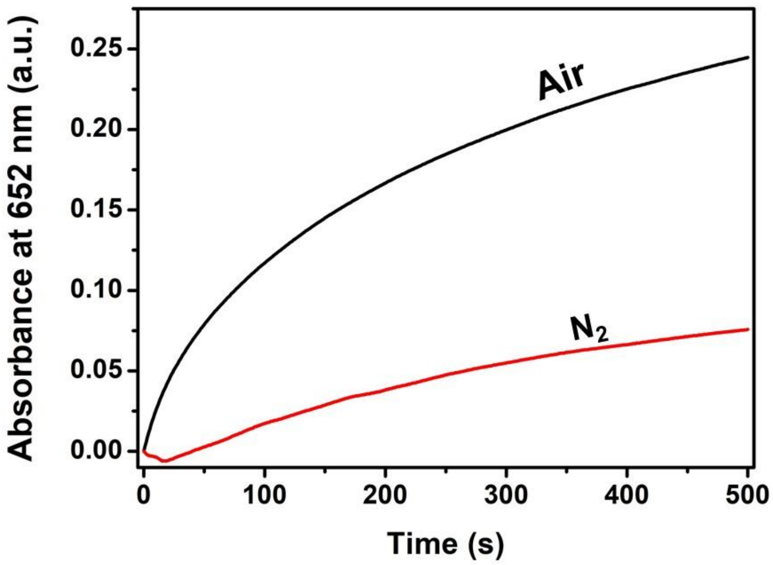

Figure S5. Influence of O_2 on the oxidase–like response of the Au@Pt nanozyme versus TMB: Evolution of the maximum absorbance peak at 652 nm at different reaction times in the presence (black line) or absence (red line) of O_2.

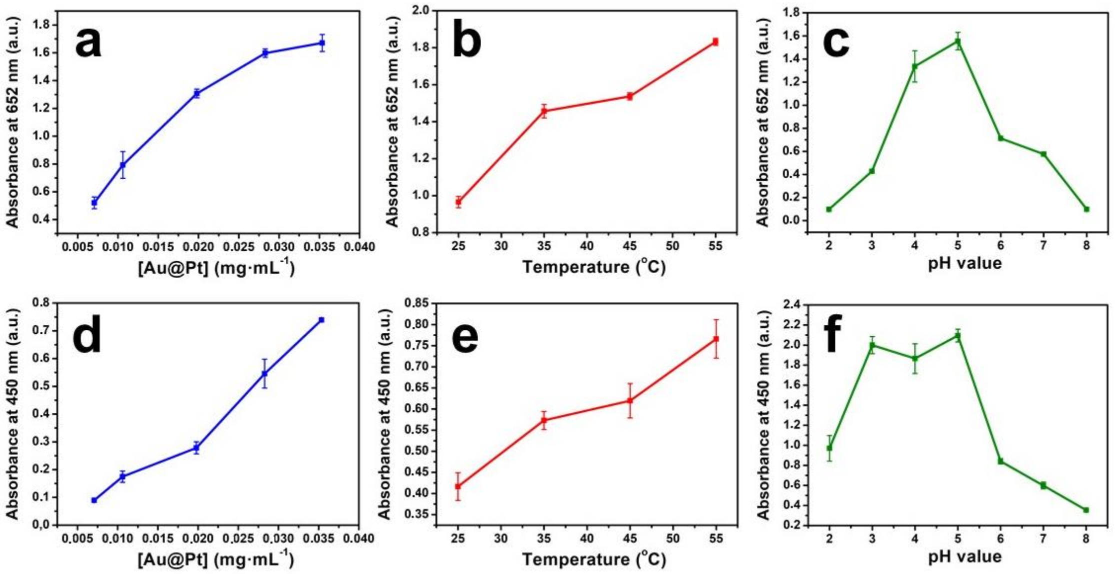

Figure S6. Influence of different parameters in the oxidase-like response of the Au@Pt nanozyme: (**a**,**d**) Nanozyme concentration; (**b**,**e**) temperature; (**c**,**f**) and pH for TMB (row from a to c) and OPD (row from d to f); Experimental conditions: Reaction time = 10 minutes. Temperature value for Figure S6$_{a,c,d,f}$ = 25 °C; pH for Figure S6$_{a,b,d,e}$ = 4.0 (adjusted with CH_3COOH/CH_3COONa buffer) and Au@Pt concentration for Figure S6$_{b,c,e,f}$ = 0.02 mg×mL^{-1}.

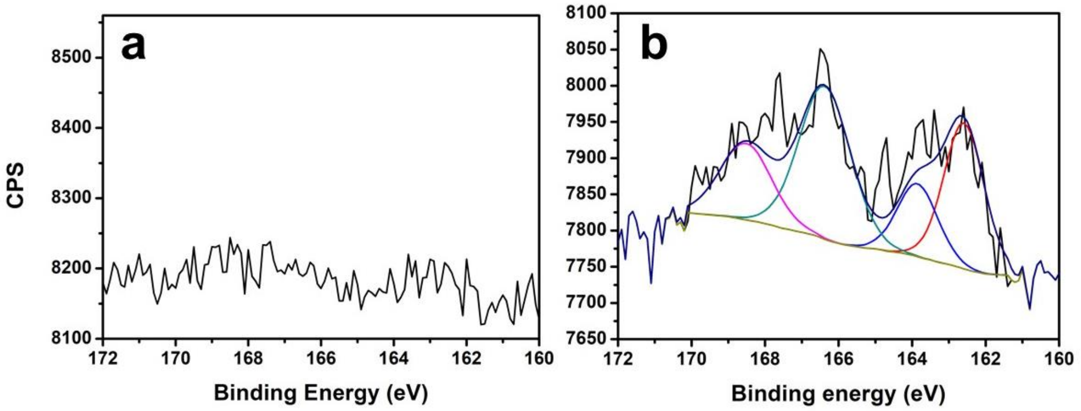

Figure S7. X-ray photoemission spectra of the S2p region (**a**) before and (**b**) after incubation of Au@Pt with GSH. The atomic percentage of S on the surface increased up to 2.5%.

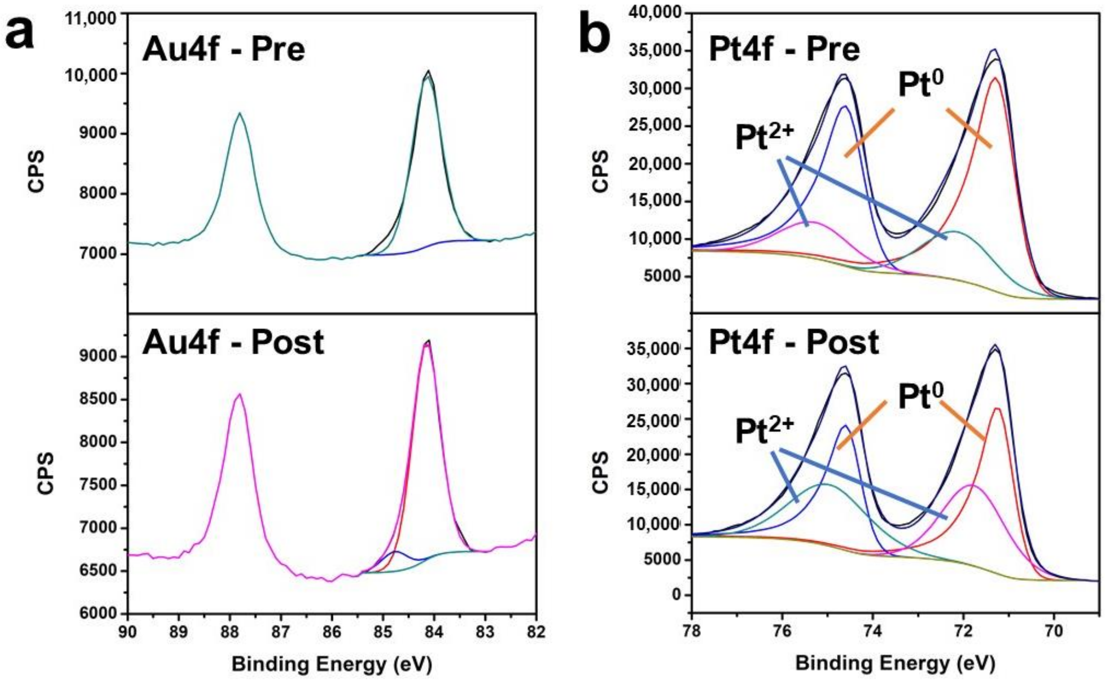

Figure S8. X-ray photoemission spectra of the (**a**) Au4f and (**b**) Pt4f regions before and after the incubation of Au@Pt nanozyme with GSH.
